# Variable-Intensity Simulated Team-Sport Exercise Increases Daily Protein Requirements in Active Males

**DOI:** 10.3389/fnut.2017.00064

**Published:** 2017-12-21

**Authors:** Jeffrey E. Packer, Denise J. Wooding, Hiroyuki Kato, Glenda Courtney-Martin, Paul B. Pencharz, Daniel R. Moore

**Affiliations:** ^1^Faculty of Kinesiology and Physical Education, University of Toronto, Toronto, ON, Canada; ^2^Frontier Research Laboratories, Institute for Innovation, Ajinomoto Co., Inc., Kawasaki, Japan; ^3^Research Institute, Hospital for Sick Children, Toronto, ON, Canada; ^4^Department of Nutritional Sciences, University of Toronto, Toronto, ON, Canada; ^5^Department of Pediatrics, University of Toronto, Toronto, ON, Canada

**Keywords:** amino acid metabolism, exercise, protein requirement, muscle, protein synthesis, athlete, recovery

## Abstract

Protein requirements are generally increased in strength and endurance trained athletes relative to their sedentary peers. However, less is known about the daily requirement for this important macronutrient in individuals performing variable intensity, stop-and-go type exercise that is typical for team sport athletes. The objective of the present study was to determine protein requirements in active, trained adult males performing a simulated soccer match using the minimally invasive indicator amino acid oxidation (IAAO) method. After 2 days of controlled diet (1.2 g⋅kg^−1^⋅day^−1^ protein), seven trained males (23 ± 1 years; 177.5 ± 6.7 cm; 82.3 ± 6.1 kg; 13.5% ± 4.7% body fat; 52.3 ± 5.9 ml O_2_⋅kg^−1^⋅min^-1^; mean ± SD) performed an acute bout of variable intensity exercise in the form of a modified Loughborough Intermittent Shuttle Test (4 × 15 min of exercise over 75 min). Immediately after exercise, hourly meals were consumed providing a variable amount of protein (0.2–2.6 g⋅kg^−1^⋅day^−1^) and sufficient energy and carbohydrate (6 g⋅kg^−1^⋅day^−1^). Protein was provided as a crystalline amino acids modeled after egg protein with the exception of phenylalanine and tyrosine, which were provided in excess to ensure the metabolic partitioning of the indicator amino acid (i.e., [1-^13^C]phenylalanine included within the phenylalanine intake) was directed toward oxidation when protein intake was limiting. Whole body phenylalanine flux and ^13^CO_2_ excretion (F^13^CO_2_) were determined at metabolic and isotopic steady state from urine and breath samples, respectively. Biphasic linear regression analysis was performed on F^13^CO_2_ to determine the estimated average requirement (EAR) for protein with a safe intake defined as the upper 95% confidence interval. Phenylalanine flux was not impacted by protein intake (*P* = 0.45). Bi-phase linear regression (*R*^2^ = 0.64) of F^13^CO_2_ resulted in an EAR and safe intake of 1.20 and 1.40 g⋅kg^−1^⋅day^−1^, respectively. Variable intensity exercise increases daily protein requirements compared to the safe intake determined by nitrogen balance (0.83 g⋅kg^−1^⋅day^−1^) and IAAO (1.24 g⋅kg^−1^⋅day^−1^) but is within the range (i.e., 1.2–2.0 g⋅kg^−1^⋅day^−1^) of current consensus statements on general recommendations for athletes.

**Clinical Trial registration:**

This trial was registered June 18, 2015 at http://clinicaltrials.gov as NCT02478814.

## Introduction

Dietary protein is important for optimal recovery from and adaptation to all types of exercise due to its ability to provide substrates for the repair and remodeling of muscle and body proteins (1, 2). The importance of this macronutrient is exemplified by the consensus statements from the American College of Sports Medicine (ACSM) identifying a target intake range of 1.2–2.0 g⋅kg^−1^⋅day^−1^ for active and athletic populations (3). However, these broad recommendations are primarily based on studies investigating the protein requirements of dichotomous strength (e.g., bodybuilding, resistance training) and endurance athletes and may not be directly translatable to athletes who fall within this continuum, such as those engaged in variable-intensity exercise.

Intermittent variable-intensity sports (e.g., basketball, soccer, ice hockey) have a unique activity pattern that incorporate characteristics of both aerobic and resistance exercise. For example, despite featuring periods of high intensity sprinting, these sports, similar to traditional more steady-state aerobic exercise, have a high reliance on aerobic metabolism (4). While there is a predominant reliance on carbohydrates for fuel during high-intensity exercise, aerobic exercise is also associated with an increase in the oxidation of amino acids (5), which may be accentuated during the latter stages of variable-intensity training or competition when glycogen availability becomes limited (6, 7). Alternatively, weight-bearing variable-intensity sports also incorporate rapid changes in velocity and direction (e.g., stop-and-go movements) that require high force production (during deceleration and acceleration), which may be more broadly aligned with that of dynamic strength or power exercises (8). Similar to high-force resistance exercise, variable speed, high-intensity “stop-and-go” type sports can also increase markers of muscle damage and induce decrements in performance (9). Nevertheless, despite the importance of dietary protein for replenishing exercise-induced oxidative losses and supporting the repair and remodeling of body (and especially muscle) proteins following exercise (1, 10), the impact of variable speed, high-intensity exercise on protein requirements, to our knowledge, have yet to be systematically investigated.

The majority of research identifying protein needs in active and athletic populations utilize the nitrogen balance technique, which has practical and analytical limitations (11, 12). Our group previously used the minimally invasive indicator amino acid oxidation (IAAO) method to demonstrate that protein requirements in endurance athletes are ~50% greater than sedentary individuals (13), which is consistent with the generally greater recommendations for active individuals estimated using nitrogen balance methodology (5, 14). Importantly, the IAAO method identifies the protein requirement that maximizes whole body protein synthesis (15), which would be important for an athlete aiming to enhance their recovery from and potentially adaptation to an exercise stimulus beyond merely maintaining nitrogen equilibrium (16, 17). Therefore, the objective of the current study was to prospectively investigate, for the first time, dietary protein needs of male athletes engaging in variable intensity exercise using IAAO methodology. We hypothesized that the safe protein intake would be greater than the current recommendations for non-active individuals determined by NBAL (0.83 g⋅kg^−1^⋅day^−1^) (18) and IAAO (1.2 g⋅kg^−1^⋅day^−1^) (12) but within ACSM commendations (1.2–2.0 g⋅kg^−1^⋅day^−1^) (3).

## Materials and Methods

### Ethics Statement

All participants were informed of the purpose of the study, the experimental procedures, and all the potential risks involved. This study was carried out in accordance with the Declaration of Helsinki with written informed consent from all subjects. The protocol was approved by the University of Toronto Health Sciences Research Ethics Board.

### General Protocol

Seven active, trained males were recruited (Table 1). Before beginning the studies, a Physical Activity Readiness Questionnaire (PAR-Q+) (19) was used to assess any health risks prior to participant enrollment in the study. Training status was characterized using the International Physical Activity Questionnaire for use with young and middle aged adults (15–69 years) and predicted VO_2max_ test [Leger Multistage Fitness Test (20)] with inclusion criteria of ≥45 min⋅day^−1^ on 5 day⋅week^−1^ moderate-vigorous activity and predicted VO_2max_ ≥50 ml O_2_·kg^−1^⋅min^−1^, respectively.

**Table 1:** Participant characteristics.

Age (years)	23 ± 1
Height (cm)	177.5 ± 6.7
Weight (kg)	82.3 ± 6.1
FFM (kg)	71.1 ± 5.5
Percent body fat (%)	13.5 ± 4.7
VO_2max_ (ml O_2_⋅kg^−1^⋅min^−1^)	52.3 ± 5.9
Habitual energy expenditure (kcal⋅day^−1^)	4,311 ± 297
MPVA (min⋅day^−1^)	239 ± 72
Daily steps	14,270 ± 3,340

*FFM, fat-free mass; MVPA, minutes of moderate to vigorous physical activity (≥3 metabolic equivalents). Means ± SD*.

The study design was based on the minimally invasive IAAO model used in healthy adults (12) and children (21) after pilot studies were performed to ensure stability of background ^13^CO_2_ and carbon dioxide production after an acute bout of exercise (Data Sheet S1 in Supplementary Material). In a randomized fashion, participants subject completed 4–10 trials that included a 2-day controlled diet prior to a metabolic trial day. The controlled diet consisted of commercially available, pre-packaged, or frozen foods that provided 1.2 g⋅kg^−1^⋅day^−1^ of protein (in accordance with the current recommended protein intake for endurance and resistance trained athletes) (3) and sufficient energy to match the habitual energy expenditure as previously measured during a habitual 3-day accelerometer record. Each metabolic trial involved the performance of a controlled exercise stimulus. Study days consisted of two components: a modified version of the Loughborough Intermittent Shuttle Test (LIST) and a subsequent 8-h metabolic trial that provided constant energy and a variably protein intake (see below for details). Each trial was separated by at least 4 days.

### Metabolic Trial Day

**Figure 1:**
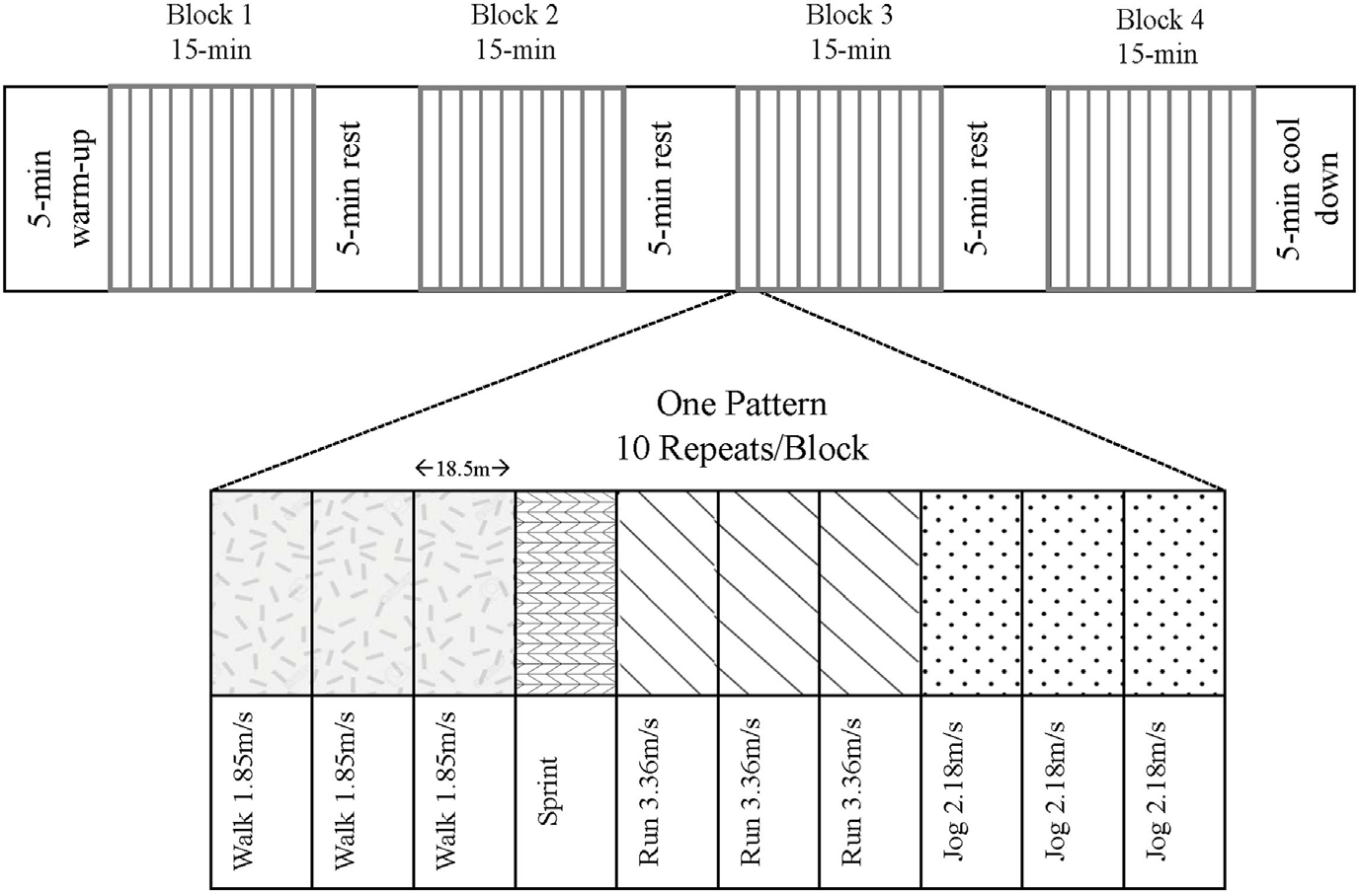
The modified Loughborough Intermittent Shuttle Test. Subjects followed an audio prompt to complete the variable-intensity pattern 10 times per block. There were four blocks separated by 5-min breaks, totaling 75-min of exercise plus a 5-min warm-up and cool-down at a self-selected pace.

Following an overnight fast, participants consumed a protein-free liquid carbohydrate beverage [1.0 g carbohydrate·kg^−1^⋅day^−1^ as a 1:1 ratio of maltodextrin (Polycal^®^; Nutricia, Amsterdam, Netherlands) and sports drink powder (Gatorade^®^ Endurance Formula; PepsiCo, Purchase, NY, USA] before reporting to the laboratory. The purpose of this beverage, which was consumed 1 h prior to the exercise, was to help replenish liver glycogen and provide some exogenous carbohydrate to fuel the variable-intensity exercise. Subjects partook in the modified LIST, which consisted of repeated 18.5-m shuttle runs resembling play in organized sports such as rugby and soccer (22, 23). The modified LIST was audio-prompted and comprised of four segments of 15 min of variable-intensity exercise including sprinting, running (90% VO_2_-max speed), jogging (60% VO_2_-max speed), and walking paces (Figure 1). Intensities were previously determined from the speed obtained at a level 12 on the beep test (4.0 m/s), which approximated the average VO_2max_ of our population. The total time commitment for the LIST exercise stimulus was approximately 75 min (4 × 15 min blocks of variable-intensity exercise + 3 × 5 min of rest between blocks).

Upon completion of the modified LIST, subjects received the study diet containing a randomly assigned protein intake (0.2–2.6 g⋅kg^−1^⋅day^−1^) as eight isocaloric and isonitrogenous hourly meals each providing one-twelfth of the participant’s total daily energy requirement (Table 2). The study diet was provided in the form of protein-free cookies (24) and test drinks, the latter of which contained protein-free powder (PFD-1; Mead Johnson, Evansville, IN, USA), flavoring crystals (Tang; Kraft, Don Mills, Canada), grape seed oil, maltodextrin (Polycal^®^), and a crystalline amino acid mixture (Ajinomoto North America, Inc., Raleigh, NC, USA). The amino acid composition of the test protein intake resembled that of egg protein with the exception of phenylalanine and tyrosine, which were held constant at an intake of 30.5 and 40.0 mg⋅kg^−1^⋅day^−1^, respectively (12, 13). The inclusion of excess tyrosine is to ensure metabolic partitioning of the phenylalanine carboxyl carbon toward protein synthesis or oxidation during stable isotope ingestion (21, 25). The study diet energy intake was calculated as:

$$\left(\text{REE}\times 1.5\right)+\left[\left(0.1425_{\text{(kcal/kg/min)}}\times {\text{Weight}}_{\text{(kg)}}\times 75_{\text{(min)}}\right)\times 1.1\right],$$

where REE = Resting Energy Expenditure During Sleep (kcal) (recorded using the Sensewear Body Media Armband Accelerometer) (26); 1.5 = activity factor (to account for the active nature of the participants and to be consistent with previous IAAO studies in rested adults) (12); 0.1425 = average energy expenditure during list exercise stimulus (kcal/kg/min); 75 min = duration of the modified list exercise stimulus; and 1.1 = 10% buffer for energy expended during the LIST exercise stimulus (to ensure that participants were in a surplus of energy and to account for individual differences in energy expenditure). As such, by providing sufficient energy intake, the protein intake derived from the experiment would be representative of a true protein requirement rather than an overestimation to partly meet energy needs. A priming dose of NaH^13^CO_3_ (0.176 mg·kg^−1^; CIL Canada, Inc., Montreal, QC, Canada) and l-[1-^13^C] phenylalanine (1.86 mg·kg^−1^; Cambridge Isotopes Laboratories, Tewksbury, MA, USA) was ingested in the fifth test drink (12). All subsequent test drinks during the metabolic trial included 1.20 mg·kg^−1^of l-[1-^13^C]phenylalanine as part of the total intake to maintain isotopic steady state until the end of the metabolic trials.

**Table 2:** Protein intake and phenylalanine flux by participant.

Participant	Protein intake (g kg^−1^ day^−1^)	Phenylalanine flux (μmol kg^−1^ h^−1^)
1	0.25, 0.75, 1.05, 1.15, 1.60, 1.70, 2.00	71.7 ± 6.1
2	0.225, 0.45, 0.55, 0.95, 1.30, 1.40, 1.95, 2.25, 2.55, 2.60	74.0 ± 11.1
3	0.35, 0.50, 1.0, 1.20, 2.05	102.9 ± 28.5
4	0.30, 0.60, 0.85, 1.10, 1.50, 1.90, 2.15, 2.50	62.3 ± 12.8
5	0.325, 0.925, 1.325, 2.45	65.0 ± 2.9
6	0.20, 0.65, 0.80, 1.25, 1.45, 1.80, 2.10	81.1 ± 18.2
7	0.40, 0.70, 0.90, 1.35, 1.55, 1.75, 2.20	73.7 ± 10.4

*Means ± SD*.

### Sample Collection and Analysis

Breath and urine samples were collected throughout the metabolic trial. Prior to the fifth meal, four breath samples were taken at 15-min intervals, and three urine samples were collected at 30-min intervals in order to establish baseline ^13^CO_2_ and l-[^13^C]phenylalanine enrichment, respectively (12). The rate of CO_2_ production was then measured over 20–25 min following the fifth hourly meal, but before the seventh hourly meal using indirect calorimetry (MOXUS Metabolic Cart, AEI Technologies) to determine steady state metabolism. Eight plateau breath and five plateau urine samples were collected at 15 and 30-min intervals, respectively, commencing 2 h after the onset of tracer ingestion. Breath samples were stored at room temperature until analyzed. Urine samples were stored at −20°C prior to analysis.

Enrichment of ^13^C in breath was analyzed in triplicate by continuous-flow isotope ratio mass spectrometry (20/20 isotope analyzer; PDZ Europa Ltd., Cheshire, United Kingdom). Urinary l-[1-^13^C]phenylalanine enrichment was determined by LC/MS/MS (API4000 197 triple quadrupole mass spectrometer, Applied Biosystems, Foster City, CA, USA) in positive electrospray ionization mode. Isotopic enrichment was expressed as molecule percent excess and was calculated from peak area ratios during isotopic steady state at plateau and baseline.

### Tracer Kinetics

Phenylalanine flux (PheRa, μmol⋅kg^−1^⋅h^−1^), the rate of appearance of ^13^CO_2_ in breath (F^13^CO_2_; μmol⋅kg^−1^⋅h^−1^), and phenylalanine oxidation (PheOx; μmol⋅kg^−1^⋅h^−1^) were calculated according to the stochastic model of Matthews et al. (27) as follows:

$$\text{PheRa}=\text{i}\cdot \left(\frac{E_{\text{i}}}{E_{\text{u}}}\right)-I,$$

where i is the rate of l-[1-^13^C] phenylalanine ingested (μmol⋅kg^−1^⋅h^−1^), *I* is the rate of l-phenylalanine ingested (μmol⋅kg^−1^⋅h^−1^), *E*_i_ and *E*_u_ are the isotopic enrichments as mole fractions of the test drink and urinary phenylalanine, respectively, at isotopic plateau.

$$F^{13}CO_{2}=\left(V_{{\text{CO}}_{2}}\right)\cdot \left(E_{{\text{CO}}_{\text{2}}}\right)\cdot \left(44.6\right)\cdot \left(60\right)\cdot BW^{-1}\cdot \left(0.82\right)\cdot \left(100\right),$$

where $V_{{\text{CO}}_{2}}$ is the CO_2_ production rate (mL⋅min^−1^); $E_{{\text{CO}}_{\text{2}}}$ is the ^13^CO_2_ enrichment in expired breath at isotopic steady state (atom percent excess); BW is the body weight (kilograms) of the participants. The constants 44.6 (μmol⋅mL^−1^) and 60 (min⋅h^−1^) were used to convert $F_{{\text{CO}}_{\text{2}}}$ to μmol⋅h^−1^. The factor 0.82 is the correction for CO_2_ retained in the bicarbonate pool of the body in the fed state (28). PheOx was calculated using Eu as an estimate of intracellular enrichment (24, 27) as:

$$\text{PheOx}=F^{13}CO_{2}\cdot {\left(\frac{1}{Eu}-\frac{1}{Ei}\right)}^{-1}\times 100.$$

### Statistical Analysis

Unless indicated otherwise, all results are expressed as mean ± SD. Protein or specific amino acid requirements determined by the IAAO technique have been reported in non-exercising populations with a range of 35–56 trials (12, 21). A range of test protein intake has been demonstrated as a better modeling fit than seven discrete intakes (12, 21) and there is no difference between the correlation strength between 35 and 43 trials using this approach (*R*^2^ = 0.60 and 0.63, respectively). As such, we aimed to complete a comprehensive *n* = 45 metabolic trials in the present study.

A mixed linear model with the subject as a random variable by using Proc Mixed program (SAS university version; SAS Institute Japan, Tokyo, Japan) was used to analyze the effects of protein intake on F^13^CO_2_, phenylalanine flux, and phenylalanine oxidation. A biphasic linear regression crossover analysis was performed on F^13^CO_2_ to determine the average protein requirement and recommended protein intakes in agreement with previous studies (12, 21). Protein intake at the breakpoint represented the estimated average requirement (EAR) whereas the safe intake was estimated as the upper limit of 95% CI of the breakpoint.

## Results

### Phenylalanine Flux

**Figure 2:**
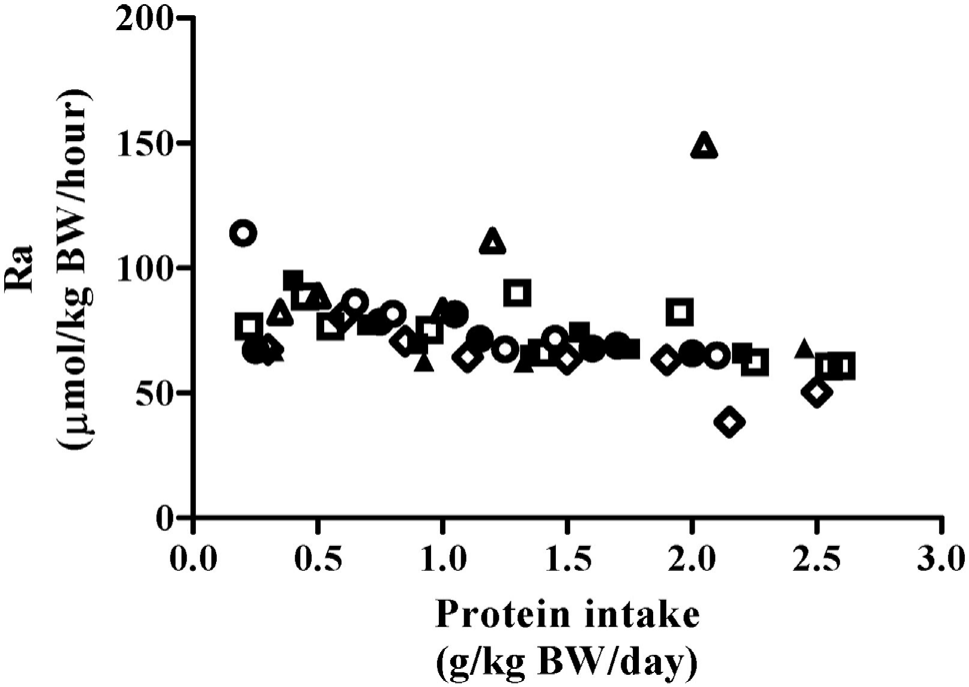
Relationship between Phe Ra and protein intake after exercise stimulus. Each data point represents PheRa of the individual metabolic trial day. Shapes represent trials performed by an individual subject. The slope of the regression line was not significantly different from 0 (*P* = 0.45).

Phenylalanine flux was not impacted by protein intake (Figure 2, *P* = 0.45) and was 75.8 ± 13.5 µmol⋅kg^−1^⋅h^−1^ when collapsed across participants and protein intakes (Table 2). Thus, the phenylalanine pool did not change in response to increasing test protein intakes, suggesting that the change in phenylalanine oxidation reflected whole-body protein synthesis.

### Average Protein Requirement and Recommended Protein Intake

**Figure 3:**
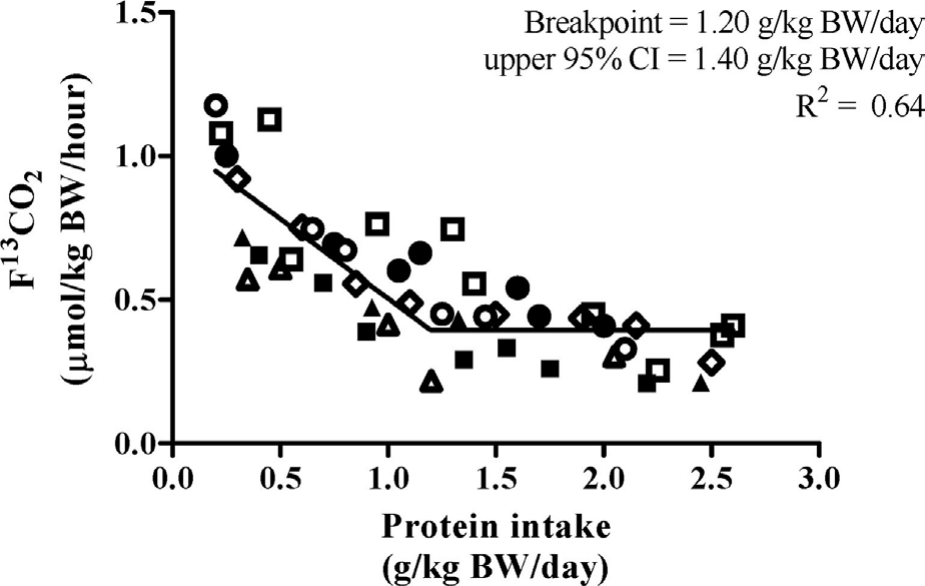
Relationship between protein intake and F^13^CO_2_. 7 participants completed 45 metabolic trials with a range of test protein intake (0.20–2.60 g⋅kg^−1^⋅day^−1^). The breakpoint estimated the average protein requirement. The breakpoint was determined by using a biphasic linear regression crossover analysis. The average protein requirement and recommended protein intake were estimated to be 1.20 and 1.40 g⋅kg^−1^⋅day^−1^, respectively (*R*^2^ = 0.64).

Biphase linear regression crossover analysis (*R*^2^ = 0.64) of F^13^CO_2_ data revealed a breakpoint at 1.20 g⋅kg^−1^⋅day^−1^ with an upper 95% CI (i.e., population-safe protein requirement) of 1.40 g⋅kg^−1^⋅day^−1^ (Figure 3). The biphase linear regression crossover analysis (*R*^2^ = 0.62) of phenylalanine oxidation revealed a breakpoint of 1.54 g⋅kg^−1^⋅day^−1^ with an upper 95% CI of 1.77 g⋅kg^−1^⋅day^−1^. F^13^CO_2_ data are generally considered to be more robust as they more closely align with rates of phenylalanine hydroxylation determined from apolipoprotein B-100 enrichment and, hence, is more reflective of the true intracellular enrichment for protein synthesis (29).

## Discussion

The aim of this study was to utilize the minimally invasive IAAO technique to determine the safe protein intake in active individuals following variable-intensity exercise that would model a stop-and-go, team sport-type (e.g., soccer) stimulus. We observed an EAR and safe protein intake (as determined by the upper 95% CI) to be 1.20 and 1.40 g⋅kg^−1^⋅day^−1^, respectively. Importantly, our 95% CI as a percentage of the EAR was similar to our previous observations in active females (i.e., ~17 vs 21%, respectively) (30) but less than studies in resistance-trained males at rest (~29%) (31) and non-exercising adults (~29%) (12), indicating our data displayed a robust and relatively homogenous bi-phase response. Although the Institute of Medicine does not consider that active individuals require a greater daily protein intake (32), our values exceed the current EAR (0.66 g⋅kg^−1^⋅day^−1^) and safe intake (0.83 g⋅kg^−1^⋅day^−1^) determined by the NBAL technique (18) and were numerically greater than those requirements established by the IAAO technique in non-exercised individuals (0.93 and 1.2 g⋅kg^−1^⋅day^−1^, respectively) (12). These results are consistent with the current broad (i.e., 1.2–2.0 g⋅kg^−1^⋅day^−1^) sports nutrition consensus recommendations based on primarily NBAL studies (3) and suggest that active individuals engaged in weight-bearing, variable-intensity exercise require a greater protein intake than their non-active peers.

Phenylalanine flux in the present study (~76 μmol⋅kg^−1^⋅h^−1^) was generally greater than that of non-exercising adults (~59 μmol⋅kg^−1^⋅h^−1^) (12), which is consistent with previous athletic populations utilizing the IAAO (13, 31) and would suggest a greater whole body protein turnover. Muscle protein turnover is elevated after acute aerobic (33, 34) and resistance exercise (35). Moreover, whole body and muscle protein turnover have also been reported to be increased in trained strength (36, 37) and endurance athletes (38, 39) at rest, which could suggest the trained status of our participants contributed to a chronically elevated phenylalanine flux independent of any acute effect of exercise. Therefore, while it is unclear what potential site(s) may be responsible for this increased phenylalanine flux and/or whether it was an acute effect of exercise or a chronic adaptation to training, the lack of effect of protein intake on this variable is consistent with a robust estimation of the average and safe protein intake from F^13^CO_2_ (21).

Current recommendations for dietary protein in athletic populations are primarily based on research from the dichotomous training modalities of aerobic and resistance exercise (3, 5, 40). Moreover, the broad recommendations (i.e., 1.2–2.0 g⋅kg^−1^⋅day^−1^) from consensus statements such as the ACSM (3) are generally nonspecific, which perhaps reflects the multitude of factors that may influence requirements (e.g., exercise modality, energy status, sex, etc.) and, in the case of team sport-type athletes, the limited research on athlete sub-groups. We demonstrate that athletes who engage in variable-intensity, stop-and-go exercise characteristic of many team sports (e.g., soccer, hockey, rugby, etc.) have an increased protein requirement relative to the EAR for non-exercised individuals based primarily on nitrogen balance data (i.e., 1.2 vs 0.66 g⋅kg^−1^⋅day^−1^) (18). In addition, the safe intake in the present study was numerically greater than an IAAO-derived requirement in non-exercising adults (i.e., 1.2 g⋅kg^−1^⋅day^−1^) (12). Our results contribute to the growing body of research demonstrating the amount and type of physical activity has a modifiable effect on daily protein requirements, which currently is not reflected in general nutrition guidelines (32). Nevertheless, our protein requirement is within the range of current sports nutrition consensus statements (3). With the assumption that dietary amino acids can be used to support protein synthesis or be irreversibly oxidized, development of the IAAO model has demonstrated that hepatic protein synthesis is the reciprocal of F^13^CO_2_ (41). We have also demonstrated that the synthesis of plasma albumin (a hepatic export protein) mirrors that of muscle protein synthesis during recovery from exercise (42). Collectively, these data suggests that team sport athletes who adhere to the current safe dietary protein intake, as most generally do when consuming adequate energy (43, 44), would obtain a sufficient quantity of this important macronutrient to maximize whole body protein synthesis to support their recovery from and/or adaptation to their sport-specific training. However, it may be relevant to highlight that the present safe intakes were derived from the consumption of a high quality, reference egg protein and may not directly translate to diets based solely or predominantly on plant-based sources (45), which may be deficient in one or more essential amino acids (46). Thus, athletes, who if omnivorous may obtain greater than ~40% of the dietary protein from plant-based sources (43), should ensure they consume complementary plant-based proteins (e.g., grains and legumes) or a combination of high quality plant and animal-based sources to ensure the current safe intake is met with nutritionally complete dietary protein(s) (45). Although it remains to be determined whether the present requirement represents an “optimal” intake from the standpoint of maximizing athlete performance (16, 17), the protein intake determined herein could be viewed as minimum target for athletes engaged in variable-intensity, weight-bearing exercise.

The moderately greater requirements from the present study relative to a non-exercised population is ultimately due to the incorporation of the exercise stimulus (12). The variable intensity, stop-and-go nature of the LIST test mimics the physiological demands of a soccer match and can increase markers of exercise-induced muscle damage (47), which would ultimately stimulate skeletal muscle remodeling to facilitate post-exercise recovery (10). It is possible that this adaptive remodeling contributed to the elevated rates of phenylalanine flux and the greater protein requirements in the present study compared to a non-exercised population (12). Alternatively, the metabolic demands of the exercise stimulus [i.e., ~70% VO_2max_ (4, 23)] would have also increased amino acid oxidation (48). Assuming a contribution of ~5% toward total energy expenditure, the LIST could have resulted in ~0.13 g⋅kg^−1^ of protein oxidized over the 75-min exercise period. This increased oxidative loss could potentially explain ~65% of the difference between the EAR determined herein and previous non-exercised population estimates by IAAO (12). These oxidative amino acid losses have previously been suggested to contribute to the greater protein requirements of strength athletes (i.e., football and rugby) who included sprint training in their programs (37). Therefore, while we are unable to determine what specifically contributed to the moderately elevated protein requirement in our athletes, our results with variable-intensity exercise are consistent with well-established research highlighting the physiological demands of endurance and resistance exercise modalities increase the metabolic requirement for this important macronutrient (5, 40). Additional research on lower impact weight bearing sports (e.g., ice hockey) and/or training modalities that include both resistive and aerobic components (e.g., concurrent training, crossfit) could be conceivable future avenues of study to advance our understanding of the nutritional requirements that span the classic strength-endurance continuum of exercise demands. Moreover, the suggested benefit of protein supplementation to improve exercise performance and anaerobic adaptations during training programs (49) could be suggestive of a slightly elevated protein requirement during consecutive days of training or competition (e.g., tournament play) for team sport athletes. Inasmuch as the oxidative losses were the primary determinant of the increased demand, our results could subsequently suggest that protein requirements may be lower on a non-training day for similar team sport athletes. Alternatively, team sports that require lower aerobic metabolism and/or include additional breaks in play (e.g., American football) might have proportionally lower protein requirements on a single day of exercise. Ultimately, the array of variables that may encompass variable intensity team sport-type athletes and their exercise demands would require additional research to titrate the impact of each on protein requirements in this population. Thus, we posit that the present results provide the background to which the impact that additional exercise/training variables may have on protein requirements of team sport can be compared.

In summary, we report that male athletes engaged in weight-bearing team sport-like variable intensity exercise have an estimated average protein requirement (1.2 g⋅kg^−1^⋅day^−1^) and safe intake (1.40 g⋅kg^−1^⋅day^−1^) that are greater than the current safe intake (estimated by NBAL) (18) and by IAAO in non-exercised males (12). Provided athletes adhere to current sports nutrition guidelines and consume adequate energy (3), these minimum requirements would likely be satisfied with a complete diet containing high quality and/or complementary protein sources.

## Ethics Statement

All participants were informed of the purpose of the study, the experimental procedures, and all the potential risks involved. This study was carried out in accordance with the Declaration of Helsinki with written informed consent from all subjects. The protocol was approved by the University of Toronto Health Sciences Research Ethics Board.

## Author Contributions

DM, GC-M, PP designed the study. JP, DW, and HK collected and analyzed the data. DM and JP drafted the manuscript and all authors were responsible for revising its intellectual content. All authors read and approved the final manuscript.

## Conflict of Interest Statement

HK is an employee of the study funder (Ajinomoto Co., Inc.) but has no competing financial interests. All other authors have no competing interests to declare. The reviewer BW and the handling Editor declared their shared affiliation.

## Abbreviations

ACSM, American College of Sports Medicine; BW, body weight; CI, confidence interval; CO_2_, carbon dioxide; EAR, estimated average requirement; $E_{{\text{CO}}_{\text{2}}}$, ^13^CO_2_ enrichment; I, ingested l-phenylalanine; *E*_i_, test drink isotopic enrichment; *E*_u_, urinary phenylalanine enrichment; F^13^CO_2_, [^13^C]carbon dioxide excretion; IAAO, indicator amino acid oxidation; LC/MS/MS, liquid chromatography tandem mass spectrometer; LIST, Loughborough Intermittent Shuttle Test; NBAL, nitrogen balance; PheOx, phenylalanine oxidation; PheRa, phenylalanine flux; REE, resting energy expenditure; $V_{{\text{CO}}_{2}}$, carbon dioxide production; VO_2max_, maximal oxygen uptake.
