# Supplementary material for: Variable-Intensity Simulated Team-Sport Exercise Increases Daily Protein Requirements in Active Males
Source: Front Nutr. 2017 Dec 21;4:64. doi: 10.3389/fnut.2017.00064 (PMC5742662; doi:10.3389/fnut.2017.00064)
Supplement: Supplementary file 1 [file Data_Sheet_1.DOCX]

**SUPPLEMENTAL FILE: Pilot study**

**PURPOSE**

The purpose of this pilot study was to determine whether our participants reach isotopic- and metabolic- steady state (i.e. plateau in breath ^13^CO_2_ enrichment and VCO_2_ production) without a prior adaptation period. Although a previous study demonstrated that no adaptation was needed, that study did not employ an exercise stimulus prior to feeding [1]. Therefore, we examined the trend of ^13^CO_2_ enrichment and VCO_2_ production following our modified version of the Loughborough Intermittent Shuttle test (LIST, described in detail in main study document), using hourly meals with identical composition as the main study, except without addition of tracer.

**METHODS**

Three healthy, young adult male subjects (age 22.7 +/- 1.5 years; height 181.3 +/- 3.5 cm; weight 85.7 +/- 9.3 kg) reported to the laboratory after an overnight fast and having abstained from alcohol and caffeine consumption for 24-hours. Upon arrival, participants completed a 15-20 minute baseline VCO_2_ collection MOXUS Metabolic Cart (AEI Technologies) prior to consuming a protein-free breakfast providing 1g/kg of carbohydrates from Polycose (0.5g/kg) and Gatorade powder (0.5g/kg). After 1h of rest, participants completed a modified Loughborough Intermittent Shuttle Test (LIST), which consisted of four 15-minute periods of variable intensity exercise (walking, sprinting, running, jogging) separated by 5 minutes of rest (see manuscript for details).

Upon completion of the LIST exercise stimulus, subjects consumed 8 isocaloric and isonitrogenous hourly meals containing calories and protein equivalent to ⅔ of their daily energy expenditure, as measured on the Sensewear Body Media Armband Accelerometer, and protein intake (1.2g/kg/d), respectively (**Supplemental Figure 1**). Protein intake was provided as crystalline amino acids modeling the composition of egg protein, as previously described [2]. Breath samples were collected every 20 minutes throughout the 8-hour metabolic trial using QuinTron breath collection bags and vacuumed Sercon exertainers prior to ^13^CO_2_ analysis by isotope ratio mass spectrometry (20/20 isotope analyzer; PDZ Europa Ltd, Cheshire, United Kingdom). VCO_2_ was collected during the final 15-20 minutes of each hour throughout the 8-hour protocol.

The change in background ^13^CO_2_ enrichment from breath samples was determined using regression analysis (Graphpad Prism® 5; GraphPad Software, Inc.,La Jolla, CA). Establishment of isotopic steady state was evaluated by repeated linear regression analysis in which data points, beginning at time 0 min, were removed until a regression line with a slope not different from zero was achieved.

**RESULTS**

Changes in breath ^13^CO_2_ enrichment and VCO_2_ production following exercise and hourly meals are depicted in **Supplemental Figure 2**. The slope of the ^13^CO_2_ enrichment was not significantly different from zero (P > 0.05) at 160 min. The slope of the VCO_2_ was not significantly different from zero (P > 0.05) at any point during the trial.

**CONCLUSIONS**

Both background isotopic (^13^CO_2_) and metabolic (VCO_2_) steady state is achieved within 3h after an acute bout of exercise when consuming hourly meals. This supports the use of the IAAO technique to study protein metabolism in active males on a day in which they exercise, which is consistent with previous results in nonexercising, sedentary adults [1].

**REFERENCES**

1. Bross R, Ball RO, Pencharz PB: **Development of a minimally invasive protocol for the determination of phenylalanine and lysine kinetics in humans during the fed state.** *J Nutr* 1998, **128:**1913-1919.

2. Kato H, Suzuki K, Bannai M, Moore DR: **Protein Requirements Are Elevated in Endurance Athletes after Exercise as Determined by the Indicator Amino Acid Oxidation Method.** *PLoS One* 2016, **11:**e0157406.

**
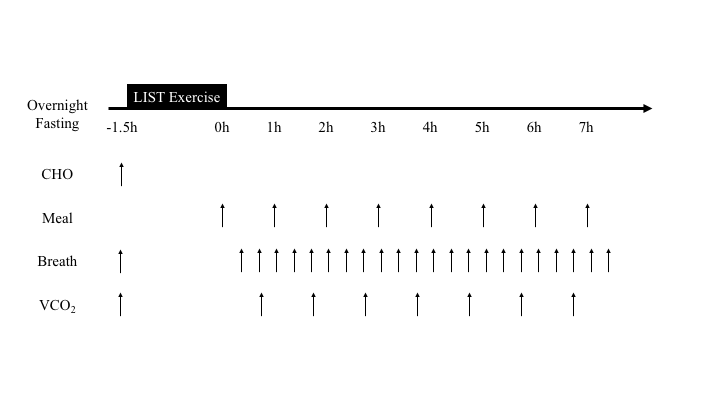
**

**FIGURE 1.** Trial day schematic. LIST = Loughborough Intermittent Shuttle Test; CHO = 1g/kg carbohydrate beverage; Meal = hourly meal providing 1/12^th^ of daily energy and protein intake; VCO_2_ = 20min carbon dioxide production.


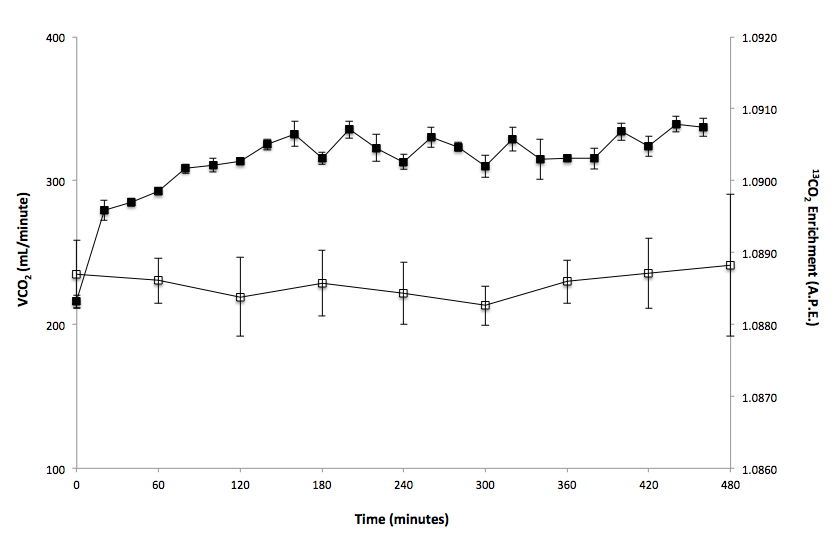


**FIGURE 2.** The effect of experimental diet on ^13^CO_2_ enrichment expressed as atoms percent excess (A.P.E.; ) and on the rate of CO_2_ production (VCO_2_; ☐). A plateau (i.e. not different from zero, *P* > 0.05) in averaged ^13^CO_2_ enrichment and averaged VCO_2_ was achieved for all subjects beginning at 180 min and 60 min respectively. Time of 0 equates to baseline ^13^CO_2_ enrichment and baseline VCO_2_.
